# *Delta-like canonical Notch ligand 1* is predictive for sepsis and acute kidney injury in surgical intensive care patients

**DOI:** 10.1038/s41598-022-17778-9

**Published:** 2022-08-03

**Authors:** Emmanuel Schneck, Fabian Edinger, Florian Uhle, Melanie Markmann, Andreas Hecker, Markus A. Weigand, Michael Sander, Christian Koch

**Affiliations:** 1 Department of Anesthesiology, Operative Intensive Care Medicine and Pain Therapy, University Hospital of Giessen and Marburg, Rudolf-Buchheim-Strasse 7, 35392 Giessen, Germany; 2 German Center of Infection Research (DZIF), Partner Site Giessen/Marburg/Langen, Giessen, Germany; 3 Department of Anesthesiology, Heidelberg University Hospital, Heidelberg, Germany; 4 Department of General and Thoracic Surgery, University Hospital of Giessen and Marburg, Giessen, Germany

**Keywords:** Diagnostic markers, Predictive markers

## Abstract

The early identification of sepsis in surgical intensive care patients is challenging due to the physiological postoperative alterations of vital signs and inflammatory biomarkers. Soluble Delta-like protein 1 (sDLL1) may be a potential discriminatory biomarker for this purpose. For this reason, this study aimed to evaluate sDLL1 for the identification of sepsis in a cohort of surgical intensive care patients. This study comprises a secondary analysis of a prospective observational study including 80 consecutive patients. The study groups included 20 septic shock patients, 20 patients each undergoing major abdominal surgery (MAS) and cardiac artery bypass surgery (CABG), and 20 matched control subjects (CTRL). The surveillance period was 72 h. The plasma concentration of sDLL1 was measured with ELISA. The plasma levels of sDLL1 were significantly elevated in septic patients compared to both surgical cohorts (septic vs. all postoperative time points, data are shown as median and interquartile range [IQR]; septic shock: 17,363 [12,053–27,299] ng/mL, CABG 10,904 [8692–16,250] ng/mL; MAS 6485 [4615–9068] ng/mL; CTRL 5751 [3743–7109] ng/mL; septic shock vs. CABG: *p* < 0.001; septic shock vs. MAS: *p* < 0.001). ROC analysis showed a sufficient prediction of sepsis with limited specificity (AUCROC 0.82 [0.75–0.82], sensitivity 84%, specificity 68%). The plasma levels of sDLL correlated closely with renal parameters (creatinine: correlation coefficient = 0.60, *r*^2^ = 0.37, *p* < 0.0001; urea: correlation coefficient = 0.52, *r*^2^ = 0.26, *p* < 0.0001), resulting in a good predictive performance of sDLL1 for the identification of acute kidney injury (AKI; AUCROC 0.9 [0.82–0.9], sensitivity 83%, specificity 91%). By quantifying the plasma concentration of sDLL1, sepsis can be discriminated from the physiological postsurgical inflammatory response in abdominal and cardiac surgical patients. However, sDLL1 has only limited specificity for the detection of sepsis in cardiac surgical patients, which may be explained by impaired renal function. Based on these findings, this study identifies the predictive value of sDLL1 for the detection of AKI, making it a potential biomarker for surgical intensive care patients.

*Trial registration* DRKS00013584, Internet Portal of the German Clinical Trials Register (DRKS), registration date 11.07.2018, https://www.drks.de/drks_web/navigate.do?navigationId=trial.HTML&TRIAL_ID=DRKS00013584.

## Introduction

Sepsis is a life-threatening complication of major abdominal and cardiovascular surgery and is associated with increased morbidity and mortality^1–4^. Despite great research efforts regarding innovative strategies for the management of sepsis, its early detection and the rapid initiation of treatment remain the most effective approaches to reduce sepsis-associated mortality^2^. Unfortunately, surgical patients are at risk for delayed diagnosis of sepsis because typical symptoms (e.g., fever, tachycardia, and altered mental status) are masked by postsurgical reactions. Furthermore, the established inflammatory biomarkers, such as C-reactive protein (CRP) are also altered by major abdominal or cardiovascular surgery^5,6^. To address this clinical problem, several biomarkers have been assessed but only a minority have been validated sufficiently and even fewer have been evaluated in surgical intensive care patients^5,7^. To date, no biomarker with sufficient discriminatory power for the differentiation of sepsis from postsurgical systemic inflammation has been introduced into the daily clinical routine^7,8^.

In 2019, Delta-like protein 1 (DLL1), a host-derived canonical Notch ligand, was identified as a novel diagnostic biomarker with promising results regarding its discriminatory value in this context^9^. DLL1 is part of the Delta/Jagged family of transmembrane proteins and functions as an activator of intracellular pathways during embryonic angiogenesis and hematopoiesis^10,11^. However, it has been reported that DLL1 is also associated with cancerous and infectious diseases^12–14^. In particular, the interaction of DLL1 and human monocytes during in vitro bacterial infections raised interest in its use as a biomarker for infection, and consequently, for sepsis. It has been shown that DLL1 activates a cellular immune response via notch signaling, resulting in the release of proinflammatory cytokines^14^. DLL1 is further involved in sepsis-induced endothelial damage leading to a loss of its barrier function^15^. Since the endothelium plays an important role for the recognition of pathogens but also for vascular integrity, DLL1 might contribute to the pathological host response during sepsis^16^. Even though DLL1 has not yet been specifically investigated in this context, the notch signal pathway is of reasonable interest for sepsis research because it modifies T-cell dysfunction and might be associated with sepsis-induced immunosuppression^17–19^. Moreover, the pathophysiological effect of DLL1 on human monocytes is of particular interest because these cells play a pivotal role in the development of sepsis. Hildebrand et al. recognized DLL1 as a potential discriminatory biomarker for sepsis and, therefore, quantified soluble DLL1 (sDLL1) as a surrogate for DLL1 receptor activation^9^. sDLL1 originates from the enzymatic degradation of the DLL1 receptor and its ligand and was measurable after in vitro bacterial infection. The authors could demonstrate that the diagnostic accuracy of sDLL1 for the identification of sepsis is superior compared to the established biomarkers, namely CRP, leukocytes, and procalcitonin (PCT). The results were also remarkable because the study sample consisted of heterogenous cohorts of patients suffering from systemic inflammation from different sources, such as sepsis, major surgery, or severe trauma. Nevertheless, these results have not yet been confirmed in other studies, and furthermore, sDLL1 has not been evaluated in cardiac surgical patients thus far. For this reason, this study aimed to describe the course of sDLL1 in cardiac and major abdominal surgical patients as well as in septic shock patients and healthy controls.

## Methods

### Study design

This secondary analysis is based on a single-center, prospective, observational proof-of-concept study of 80 patients^20^. The study is registered in the German Clinical Trials Register (trial code: DRKS00013584) and was approved by the local ethics committee (Justus-Liebig-University of Giessen, trial code: 86/18, amendment 3). Both the original study and this secondary analysis were performed in accordance with the Helsinki Declaration, and all methods and results are presented in accordance with the Strengthening the Reporting of Observational Studies in Epidemiology (STROBE) guidelines.

### Patient recruitment

After obtaining each patient’s informed consent, 80 consecutive patients were recruited between October 2018 and March 2019. Patients were excluded if they were pregnant or nursing, younger than 18 years, had a history of severe valvular heart, autoimmune, and/or hematological disease, had recently suffered severe trauma, or were in need of immunomodulatory medication, extracorporeal membrane oxygenation, or renal replacement therapy. While septic patients (n = 20) had to meet the Sepsis-3 criteria for septic shock, the surgical study groups included either patients undergoing coronary artery bypass graft surgery (CABG, n = 20) or major abdominal surgery (MAS, n = 20)^4,21^. All surgical patients suffered postoperatively from systemic inflammatory response syndrome (SIRS)^22^. Furthermore, a control group (CTRL, n = 20) for the septic shock patients was established by matching for age, gender, and underlying health conditions (arteriosclerosis, cancerous diseases, renal insufficiency, and diabetes mellitus).

### Sample processing

Blood was collected at onset, 24 h, and 72 h from septic shock patients, whereas the blood of subjects in the CTRL group was only drawn once. Surgical patients were investigated pre- and postoperatively, and after 24 and 72 h. Blood was originally drawn in ethylenediaminetetraacetic acid (EDTA) tubes and stored at − 80 °C. Clinical data including the inflammatory and renal laboratory parameters were obtained from the patient data management system (IMESO GmbH, Giessen, Germany).

### Quantification of sDLL1

The plasma concentration of sDLL1 was quantified using a commercially available enzyme-linked immunosorbent assay (ELISA) kit (RayBiotech Life, Inc., Norcross, USA) according to the manufacturer’s instructions. To fit measurements into the calibration curves and simultaneously reduce interfering matrix effects, all samples were diluted 1:30 (or higher if demanded by the concentration) with the supplied Assay Diluent A prior to the measurements. An ELx808 microplate reader (BioTek Instruments, Inc., Winooski, USA) was used for absorbance measurements, with a subsequent automatized calculation of concentrations by the corresponding Gen5 software (BioTek Instruments, Inc., Winooski, USA).

### Statistical analysis

While parametric data were described as mean and standard deviation, non-parametric data were expressed as median and interquartile range (IQR). The analysis of variations in plasma sDLL1 levels across different time points was performed by the repeated measures ANOVA test, followed by the Tukey HSD test for paired groups (septic shock group compared to matched controls). *p* Values < 0.05 were considered statistically significant. Potential correlations between plasma sDLL1 levels and inflammatory and renal parameters were analyzed by Pearson’s correlation coefficient. Receiver operating characteristic (ROC) curve analyses were used for the calculation of area under the ROC curve (AUCROC), sensitivity, and specificity of inflammatory and renal parameters. All data were stored in an external database (Microsoft Excel, Redmond, USA). Data were analyzed using R statistical software version 4.0.4 (2021-02-15; www.r-project.org).

### Ethics approval and consent to participate

All included patients approved to their study participation.

## Results

### Study cohort

The patient characteristics of the 80 included patients did not differ significantly regarding their age and sex (details are presented within the primary study^20^). 70% (n = 14) of the patients in the shock group, 75% (n = 15) in the CABG group, 60% (n = 12) in the MAS group, and 70% (n = 14) in the CTRL group were male. The ages (in years) of each group (expressed as the median [IQR]) were 69 [64–74] for septic shock, 70 [62–79] for CABG, 68 [54–70] for MAS, and 69 [66–74] for CTRL. The severity of sepsis was indicated by the sepsis-related organ failure assessment score (SOFA) score (SOFA at onset: 10.5 [10–12.5]; 24 h: 11.5 [8–13]; 72 h: 9 [5.5–14.5]), leading to an in-hospital-mortality rate of 35% (n = 7). Sepsis originated from intraabdominal infections in 60% (n = 12) of cases, whereas a pulmonary or urological source of infection was identified in 15% of cases (each n = 3) and a soft-tissue infection was detected in 10% (n = 2) of cases. MAS included Whipple’s procedure in 40% (n = 8) of patients, whereas open partial colectomy and esophagus resection were each performed in 20% (n = 4) of patients, and other types of MAS accounted for 20% (n = 4) of patients.

### Quantification of sDLL1

#### Group analyses

**Figure 1:**
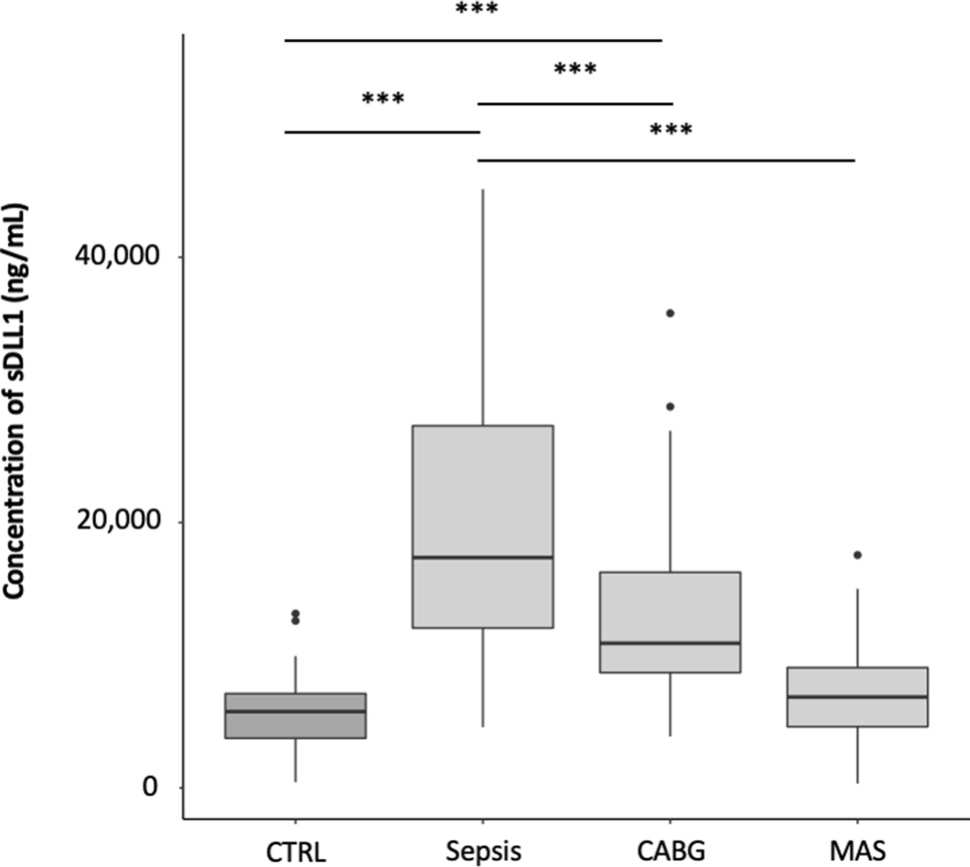
Boxplot showing the intergroup differences in plasma sDLL1 concentration. Differences with statistical significance are labeled with an asterisk (***= *p* < 0.001). *CABG* coronary artery bypass graft, *CTRL* control, *MAS* major abdominal surgery, *sDLL1* soluble delta-like canonical Notch ligand.

The amount of sDLL1 differed significantly among the study groups. The cumulative values of all time points of the septic shock patients were significantly higher than the cumulative postoperative measurements of patients undergoing CABG as well as MAS (data are shown as median and interquartile range [IQR]; septic shock: 17,363 [12,053–27,299] ng/mL, CABG 10,904 [8692–16,250] ng/mL; MAS 6485 [4615–9068] ng/mL; CTRL 5751 [3743–7109] ng/mL; septic shock vs. CABG: *p* < 0.001; septic shock vs. MAS: *p* < 0.001; Fig. 1). Although the sDLL1 concentrations of MAS patients were not significantly increased compared to the CTRL group, they were significantly elevated in septic patients and patients who underwent cardiac surgery (septic shock vs. CTRL: *p* < 0.001; CABG vs. CTRL: *p* < 0.001; MAS vs. CTRL: *p* = 0.99; Fig. 1). Compared to control subjects, the preoperative levels of sDLL1 were elevated neither in cardiac nor in abdominal surgical patients (Table 1).

**Table 1:** Overview of the inflammatory parameters within the study cohorts.

	sDLL1 (ng/mL)	Leukocytes (number/µL)	CRP (mg/mL)	PCT (µg/L)
**Control median [IQR]**	5,751 [3,743–7,109]	5.9 [5.3–7.9]	1.1 [0–6.4]	N.A
**Septic shock median [IQR]**				
Onset	21,626 [15,171–27,164]	11.9 [7.1–19.7]	229.5 [117.2–277.3]	9.2 [5.2–38.1]
24 h	21,782 [13, 20–31, 158]	13.5 [9.3–20.9]	244.6 [166.5–287.7]	10 [4 4.9–29.2]
72 h	12,959 [10,045–17,383]	14.2 [10.7–17.3]	236.5 [139.5–268.8]	7 [2.2–25.6]
**Cardiac surgery median [IQR]**				
Preoperative	7,867 [5,764–12,851]	8.1 [6.6–9.4]	3.8 [1.9–10.6]	0.2 [0.1–0.2]
Postoperative	9,688 [7,865–13,708]	11 [7.9–15]	4.3 [2.6–9.2]	N.A
24 h	12,649 [9,480–16,292]	10.7 [8.2–12.2]	75.1 [67.2–109.8]	N.A
72 h	11,485 [9,361–18,370]	10.6 [8.2–11.8]	202.4 [156.3–241.2]	1.6 [1.6]
**Major abdominal surgery median [IQR]**				
Preoperative	6,952 [3,405–8,345]	7.6 [6–9]	5.1 [1.7–10.3]	N.A
Postoperative	6,486 [4,258–8,346]	10.3 [9.4–12.5]	6.5 [2.5–11.4]	0.6 [0.4–0.7]
24 h	7,729 [6,149–9,188]	11.5 [9.3–12.9]	68 [46.6–88.5]	0.7 [0.3–0.9]
72 h	6,536 [4,179–9,603]	7.4 [6.5–11.6]	149 [115.7–200]	0.8 [0.4–0.9]

*CRP* C-reactive protein, *IQR* interquartile range, *N.A.* not available, *PCT* Procalcitonin.

#### Time courses of sDLL1

**Figure 2:**
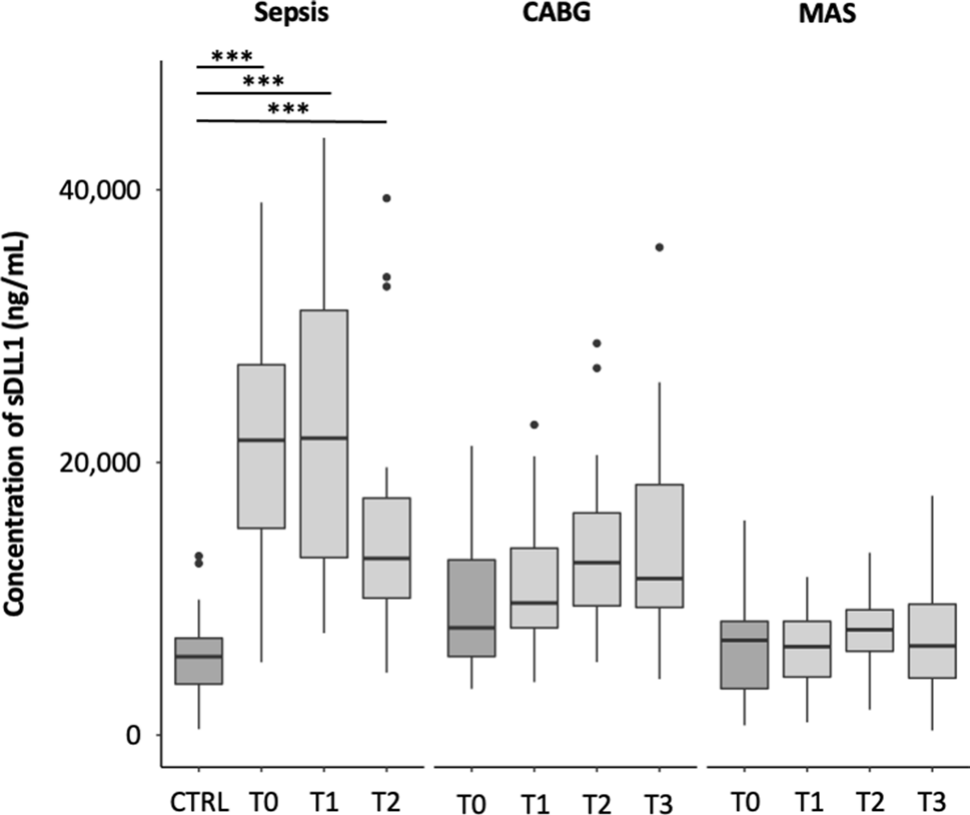
Boxplot showing the time course of plasma sDLL1 concentration in the different study groups. Differences with statistical significance are labeled with an asterisk (***= *p* < 0.001). Time points sepsis: T0 = onset, T1 = 24 h, T2 = 72 h. Time points surgical patients: T0 = preoperative, T1 = postoperative, T2 = 24 h, T3 = 72 h. *CABG* coronary artery bypass graft, *CTRL* control, *MAS* major abdominal surgery, *sDLL1* soluble delta-like canonical Notch ligand.

Septic shock patients already showed highly elevated levels of sDLL1 at onset, which persisted over 24 h and decreased non-significantly after 72 h (CTRL vs. sepsis onset: *p* < 0.001; CTRL vs. sepsis 24 h: *p* < 0.001; CTRL vs. sepsis 72 h: *p* < 0.001; Table 1; Fig. 2). In both surgical groups, the levels of sDLL1 increased, although they did not reach statistical significance in comparison to their preoperative values (Table 1; Fig. 2). Furthermore, within the first 24 h, the blood levels of sDLL1 were significantly lower in both surgical groups compared to septic patients (sepsis onset vs. CABG postoperative: *p* < 0.001; sepsis onset vs. MAS postoperative: *p* < 0.001; sepsis 24 h vs. CABG 24 h: *p* < 0.001; sepsis 24 h vs. MAS 24 h: *p* < 0.001; Table 1; Fig. 2). However, after 72 h, these differences were only detected in abdominal surgical patients (sepsis 72 h vs. CABG 72 h: *p* = 0.99; sepsis 72 h vs. MAS 72 h: *p* < 0.01; Table 1; Fig. 2). Within both surgical groups, the levels of sDLL1 did not differ between the corresponding measurement time points (Table 1; Fig. 2). Furthermore, it must be acknowledged that after 72 h, the plasma sDLL1 levels of septic patients already approached the levels of CABG patients.

### sDLL1 as potential predictive biomarker for sepsis

**Figure 3:**
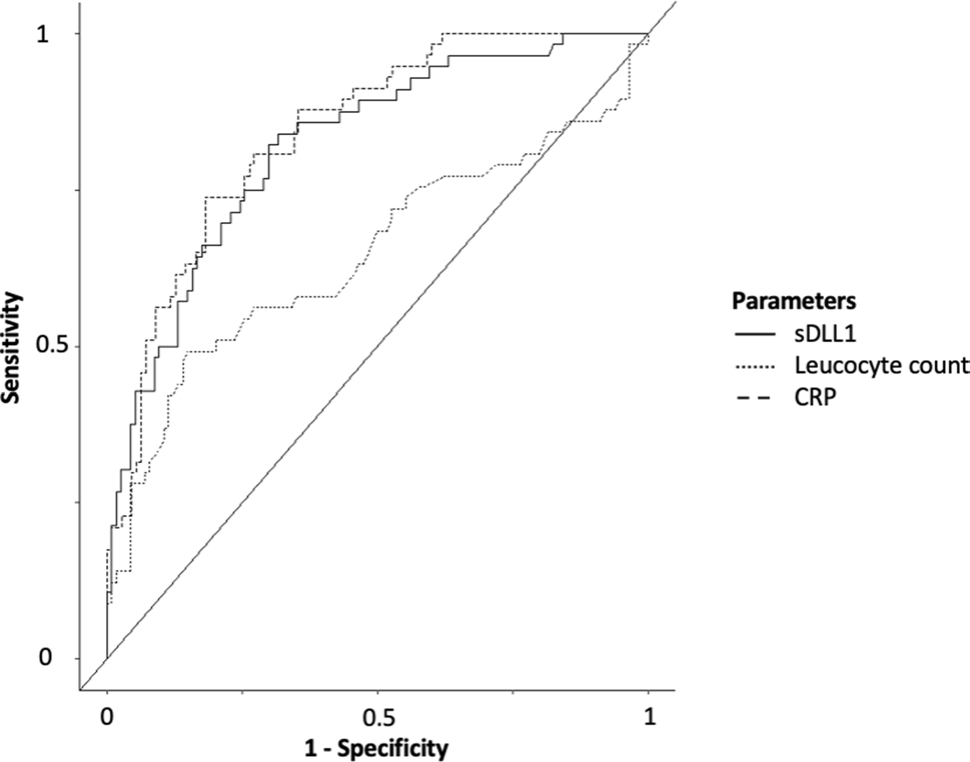
ROC analyses of the plasma levels of sDLL1 and CRP and leukocyte count at all postoperative time points and all septic time points regarding the prediction of sepsis. The corresponding values are given in Table 2. *CRP* C-reactive protein, *ROC* receiver operating characteristic, *sDLL1* soluble delta-like canonical Notch ligand.

Among surgical patients in need of intensive care, the performance of plasma sDLL1 concentration was not superior to CRP but superior to leukocyte count regarding the prediction of sepsis. The number of PCT values within the surgical cohorts was too low for ROC analysis (Fig. 3, Table 2). The plasma levels of sDLL1 correlated significantly with the SOFA score (correlation coefficient = 0.61; *r*^2^ = 0.36; *p* < 0.0001), lactate plasma concentration (correlation coefficient = 0.45; *r*^2^ = 0.20, *p* < 0.0001), and inflammatory parameters (CRP: correlation coefficient = 0.52; *r*^2^ = 0.27, *p* < 0.0001; leukocyte count: correlation coefficient = 0.29; *r*^2^ = 0.08, *p* < 0.0001; PCT: correlation coefficient = 0.29; *r*^2^ = 0.07, *p* = 0.02).

**Table 2:** Results of the ROC analysis for the prediction of sepsis. ROC analysis included all postoperative time points of the surgical patients and all time points of the septic patients.

	AUCROC [95% CI]	Cutoff value	Specificity	Sensitivity
sDLL1 (ng/mL)	0.82 [0.75–0.82]	10,633	0.68	0.84
CRP (mg/mL)	0.84 [0.78–0.84]	157.7	0.82	0.74
Leukocyte count (number/µL)	0.65 [0.55–0.65]	14.1	0.85	0.49

*AUCROC* area under the ROC curve, *95% CI* 95% confidence interval, *CRP* C-reactive protein, *ROC* receiver operating characteristic, *sDLL1* soluble delta-like canonical Notch ligand.

### sDLL1 as a potential biomarker for acute kidney injury (AKI)

**Figure 4:**
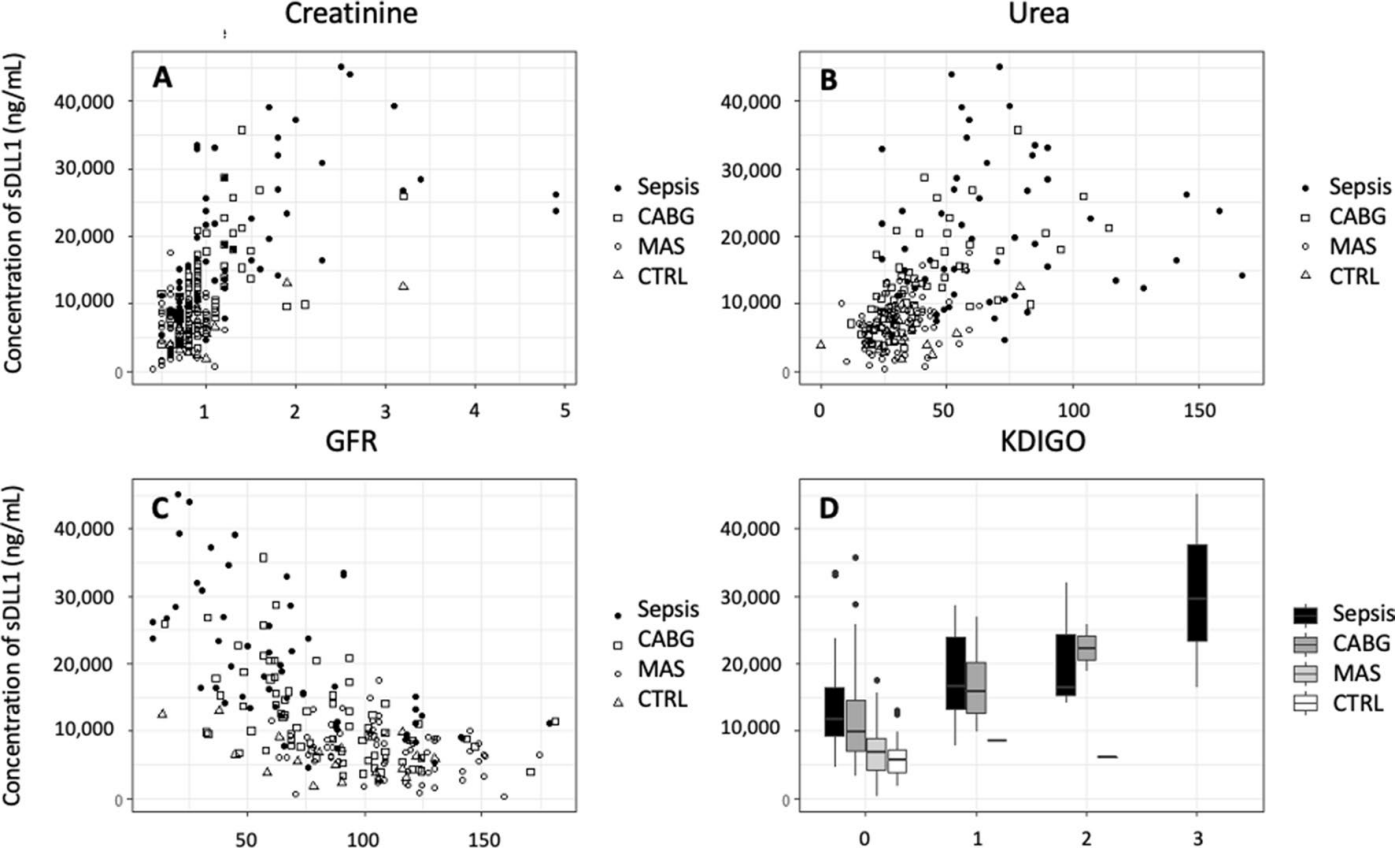
Scatterplots of sDLL1 with creatinine (**A**), urea (**B**), and GFR (**C**) as well as a boxplot (**D**) showing the concentration of sDLL1 at different KDIGO stages. *CABG* coronary artery bypass graft, *CTRL* control, *GFR* glomerular filtration rate, *KDIGO* kidney disease: improving global outcomes, *MAS* major abdominal surgery, *sDLL1* soluble delta-like canonical Notch ligand. ROC analyses of the established renal parameters regarding the prediction of AKI (defined as Kidney Disease: Improving Global Outcomes [KDIGO] stadium ≥ 1) compared to sDLL1 revealed a comparable predictive power of sDLL1 (Fig. 5, Table 3).

The plasma concentration of sDLL1 showed a strong correlation with all renal parameters (creatinine: correlation coefficient = 0.60, *r*^2^ = 0.37, *p* < 0.0001; urea: correlation coefficient = 0.52, *r*^2^ = 0.26, *p* < 0.0001; glomerular filtration rate [GFR]: correlation coefficient = − 0.61, *r*^2^ = 0.37, *p* < 0.0001; Fig. 4).

**Figure 5:**
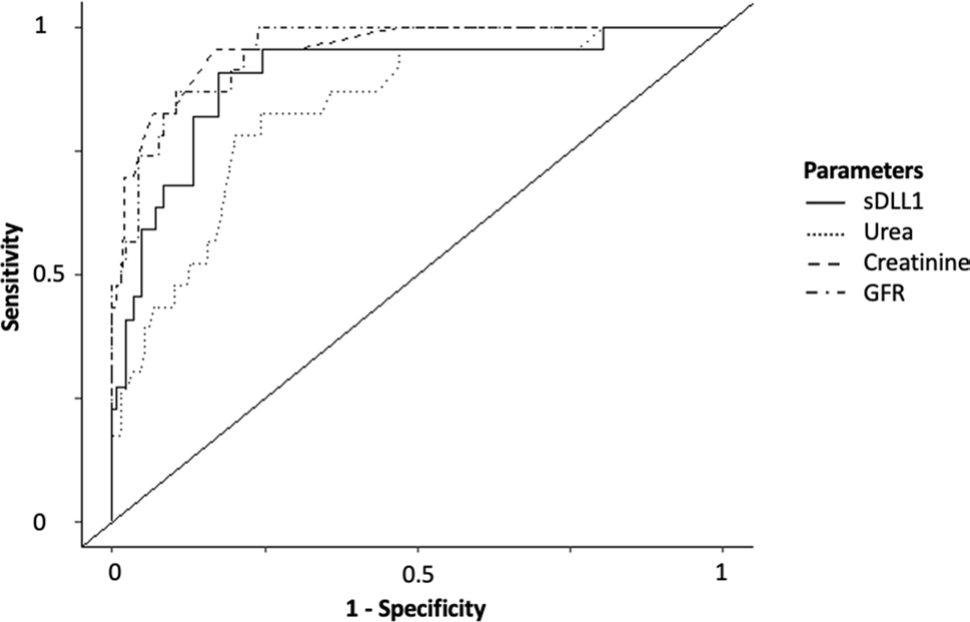
ROC analyses of the plasma levels of sDLL1, CRP, and leukocyte count of all postoperative time points and all septic time points regarding the prediction of AKI (defined as Kidney Disease: Improving Global Outcomes [KDIGO] stadium ≥ 1). The corresponding values are given in Table 3. *AKI* acute kidney injury, *CRP* C-reactive protein, *GFR* glomerular filtration rate, *ROC* receiver operating characteristic, *sDLL1* soluble delta-like canonical Notch ligand.

**Table 3:** Results of the ROC analysis for the prediction of AKI.

	AUCROC [95% CI]	Cutoff value	Specificity	Sensitivity
sDLL1 (ng/mL)	0.9 [0.82–0.9]	16,345	0.83	0.91
Creatinine (mg/dL)	0.96 [0.92–0.96]	1.15	0.83	0.96
Glomerular filtration rate (mL/min)	0.95 [0.92–0.95]	56.3	0.90	0.87
Urea (mg/dL)	0.83 [0.75–0.83]	51.5	0.76	0.83

ROC analysis included all postoperative time points of the surgical patients and all time points of the septic patients.

*AKI* acute kidney injury, *AUCROC* area under the ROC curve, *95% CI* 95% confidence interval, *ROC* receiver operating characteristic, *sDLL1* soluble delta-like canonical Notch ligand.

## Discussion

Formerly known as a biomarker in cancer research, sDLL1 was first introduced as a distinct biomarker for sepsis in a mixed cohort of intensive care patients in 2019^9^. This secondary analysis of an observational study aimed to confirm the findings of Hildebrand et al. and to evaluate the predictive value of sDLL1 for the detection of sepsis in cardiac surgical patients, which has not been investigated thus far.

First, this study showed a significant increase in plasma sDLL1 concentration in septic patients compared to surgical patients and control subjects. In patients undergoing MAS, the increase in sDLL1 level was detectable over 72 h. However, following cardiopulmonary bypass (CBP), the levels of sDLL1 increased more than in abdominal surgical patients, explaining its diminishing difference to the levels in septic patients after 24 h. Moreover, cardiac surgical patients also featured an elevated sDLL1 compared to the CTRL group, which was not observed in MAS patients. To summarize, regarding abdominal surgical patients, the results of this study confirm the findings of Hildebrand et al. while revealing that cardiac surgical patients differed from MAS patients in terms of higher sDLL1 plasma levels. To the best of our knowledge, the kinetics of the plasma concentration of sDLL1 have not yet been described in cardiac surgical patients and should therefore also be assessed in future studies. Second, the predictive value of sDLL1 for the detection of sepsis was sufficient, but it did not perform superiorly to CRP. This is mainly due to the limited specificity of sDLL1, which may be explained by the increase in sDLL1 levels in cardiac surgical patients. To summarize, these results indicate that the use of sDLL1 for discrimination between sepsis and physiological postoperative inflammatory reactions may be impaired in cardiac surgical intensive care patients, and leaves the question of how they differ from patients after MAS.

Norum et al. investigated the diagnostic value of plasma sDLL1 levels in patients suffering from dilated cardiomyopathy. Along with a good correlation of sDLL1 with disease severity and cardiac function, they identified an association with renal parameters such as creatinine and GFR^23,24^. Since cardiac surgical patients are at increased risk of renal failure, we also searched for a correlation between the plasma levels of sDLL1 and surrogate parameters of renal function to potentially explain the elevation of sDLL1 following CBP^25^. Indeed, this study was able to confirm the association of sDLL1 with renal parameters by showing a strong positive correlation between sDLL1 and the plasma creatinine and urea concentration as well as a negative correlation to the GFR. These findings indicate that CPB-induced AKI may cause increased sDLL1 plasma levels. However, despite this strong hint, the underlying causative mechanisms, such as an accumulation of sDLL1 caused by impaired renal function, have not yet been identified. Even though the interaction between sDLL1 and renal function limits its specificity for the detection of sepsis, it may be useful for the identification of AKI. With a prevalence of up to 60%, AKI represents a major burden in critically ill patients^26,27^. Even though the definition of AKI has been adapted and novel biomarkers have been introduced, its rapid identification still poses a clinical challenge. The plasma concentration of sDLL1 showed high sensitivity and specificity for the prediction of AKI stages ≥ 1 in surgical intensive care patients, independent of the presence of surgery- or CPB-induced SIRS or sepsis as the underlying condition. It performed better than urea and was comparable to creatinine and GFR. Even though novel renal biomarkers have not been investigated in this study, the high predictive value of sDLL1 is promising. For example, the *Translational Research Investigating Biomarkers and End Points for Acute Kidney Injury* (TRIBE-AKI) study investigated urine and plasma neutrophil gelatinase-associated lipocalin (uNGAL and pNGAL, respectively) in cardiac surgical patients and revealed only a moderate predictive performance of these well-investigated biomarkers (AUCROC: uNGAL: 0.67; pNGAL: 0.70)^28^. Comparable predictive capacity has also been shown in mixed cohorts of critically ill patients^29,30^. Furthermore, a recent meta-analysis reported an AUCROC of 0.75 for uNGAL for the identification of severe AKI^31^. Plasma concentration of sDLL1 performed even better than the product of tissue inhibitor of metalloproteinase 2 and insulin-like growth factor-binding protein-7 (TIMP2*IGFBP7, Nephrocheck™) that has already been approved for the prediction of AKI in critically ill patients (AUCROC: 0.74 in critically ill patients and 0.83 after cardiac surgery)^27,32–35^. However, it is worth noting that this study is the first to report the predictive value of sDLL1 in the context of renal failure. For this reason, the AUCROC cannot be directly compared to the high number of patients included in the mentioned studies and meta-analyses. Nevertheless, it justifies further research in surgical intensive care patients, especially in cardiac surgical patients. Apart from the causative mechanisms of the release of sDLL1 during renal injury, future research should focus on kinetics to evaluate sDLL1 as an early predictor of AKI.

This study has some limitations. First, due to its design as a secondary analysis of an observational study, the plasma concentration of PCT was not available for all patients, making an ROC analysis and comparison to sDLL1 unfeasible. For the same reason, it was not possible to measure the concentration of sDLL1 in urine, which would be of high interest in this context. Third, only patients suffering from septic shock were included in this study. It is also worth investigating whether the identified differences between septic and surgical patients are also present in patients with a lower degree of disease severity. Furthermore, the majority of patients suffered from an abdominal source of sepsis; however, since pneumonia is a common source of sepsis in non-surgical patients, future studies should also include patients with other origins of sepsis in order to detect potential differences. Fourth, sDLL1 identifies only bacterial infections, leaving a blind gap in the detection of viral and fungal infections^14^. Finally, as mentioned previously, this study does not provide insights into the underlying causative mechanisms of sDLL1 release in surgical intensive care patients.

## Conclusion

In summary, this study could underline the potential diagnostic value of sDLL1 for the discrimination of sepsis from surgery-induced systemic inflammation within the first 24 h after intensive care unit admission. However, for the detection of sepsis, sDLL1 exhibited only limited specificity in cardiac surgical patients, which may be explained by the close association between the impaired renal parameters and increase in sDLL1. These findings have led to the identification of the high sensitivity and specificity of sDLL1 for the detection of AKI in this study, making it an interesting option for surgical intensive care patients.

## Data Availability

The datasets used and/or analysed during the current study are available from the corresponding author on reasonable request.

## Abbreviations

AKI: Acute kidney injury
AUROC: Area under the ROC curve
CABG: Coronary artery bypass graft
CPB: Cardiopulmonary bypass
CRP: C-reactive protein
CTRL: Control
DLL1: Delta-like protein 1
EDTA: Ethylenediaminetetraacetic acid
ELISA: Enzyme-linked immunosorbent assay
GFR: Glomerular filtration rate
IQR: Interquartile range
KDIGO: Kidney disease: improving global outcomes
MAS: Major abdominal surgery
PCT: Procalcitonin
pNGAL: Plasma neutrophil gelatinase-associated lipocalin
ROC: Receiver operator curve
sDLL1: Soluble delta-like protein 1
SIRS: Systemic inflammatory response syndrome
SOFA: Sepsis-related organ failure assessment score
STROBE: Strengthening the reporting of observational studies in epidemiology
TIMP2*IGFBP7: Tissue inhibitor of metalloproteinase 2 and insulin-like growth factor-binding protein-7
TRIBE-AKI: Translational research investigating biomarkers and end points for acute kidney injury
uNGAL: Urine neutrophil gelatinase-associated lipocalin
